# Biospeckle‐characterization of hairy root cultures using laser speckle photometry

**DOI:** 10.1002/elsc.201900161

**Published:** 2020-06-02

**Authors:** Carolin Schott, Juliane Steingroewer, Thomas Bley, Ulana Cikalova, Beatrice Bendjus

**Affiliations:** ^1^ Institute of Natural Materials Technology TU Dresden Dresden Germany; ^2^ Fraunhofer Institute for Ceramic Technologies and Systems IKTS Dresden Germany

**Keywords:** *Beta vulgaris*, hairy roots, laser speckle photometry, monitoring, speckle contrast

## Abstract

Monitoring is indispensable for the optimization and simulation of biotechnological processes. Hairy roots (hr, plant tissue cultures) are producers of valuable relevant secondary metabolites. The genetically stable cultures are characterized by a rapid filamentous growth, making monitoring difficult with standard methods. This article focuses on the application of laser speckle photometry (LSP) as an innovative, non‐invasive method to characterize *Beta vulgaris* (hr). LSP is based on the analysis of time‐resolved interference patterns. Speckle interference patterns of a biological object, known as biospeckles, are characterized by a dynamic behavior that is induced by physical and biological phenomena related to the object. Speckle contrast, a means of measuring the dynamic behavior of biospeckles, was used to assess the biospeckle activity. The biospeckle activity corresponds to processes modifying the object and correlates with the biomass growth. Furthermore, the stage of the cultures’ physiological development was assessed by speckle contrast due to the differentiation between active and low active behavior. This method is a new means of monitoring and evaluating the biomass growth of filamentous cultures in real time. As a potential tool to characterize hairy roots, LSP is non‐invasive, time‐saving, can be used online and stands out for its simple, low‐cost setup.

**Abbreviations**
*B. vulgaris*: *Beta vulgaris*
hr: hairy roots
LSP: laser speckle photometry

## 1 INTRODUCTION

Plant cells and tissue culture generate a variety of valuable nutritionally, physiologically, and pharmaceutically relevant secondary metabolites. The in vitro processing of plant cells and tissue cultures is an investigative alternative to conventional techniques and a progressive means of producing a consistent quality and quantity of natural agents and additives under optimal conditions [1, 2, 3].

Research efforts have focused on the cultivation of in vitro plant tissue cultures such as hairy roots (hr). Hairy roots are transformed plant cells caused by an infection with the soil bacterium *Agrobacterium rhizogenes (A. rizogenes)*, which transfers the T‐DNA of the bacterial root‐inducing plasmid into the chromosomal plant DNA. Hairy roots are genetically stable and exhibit high growth rates and growth in the absence of plant growth regulators. The biomass growth of hairy roots is commonly classified as being in a lag phase, an exponential growth phase or a stationary phase. Hairy roots are characterized by a root‐like, filamentous structure [1, 2, 3, 4, 5].

To develop and optimize biotechnological processes, appropriate monitoring of biomass growth and product accumulation is indispensable. Efficient investigation (fewer experiments, online monitoring, non‐invasive technique) requires a technologically advanced analytical method. Due to the heterogeneous root‐like morphology of the hairy roots, common analytical methods for determining biomass growth are time consuming and not appropriate [5].

This work focuses on investigations to characterize root‐like structure of hairy root cultures of *Beta vulgaris* (*B. vulgaris*) using an innovative, non‐invasive, and rapid optical method known as laser speckle photometry (LSP).

LSP is a novel method for the nondestructive testing of applications and contactless measurement and has been developed to evaluate material damage, hardness, and porosity [6].

**FIGURE 1:**
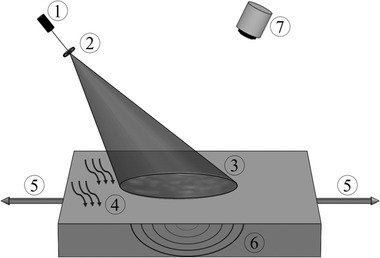
Schematic setup of laser speckle photometry. 1, Speckle excitation (laser illumination); 2, imaging optics, 3, speckle pattern; 4, thermal excitation (heating, laser); 5, mechanical excitation (load); 6, thermal distribution; 7, detector (CMOS camera)

The experimental setup of LSP is simply configured [6, 7, 8]. Figure 1 illustrates the schematic setup of LSP technology.

Basically, the experimental setup (Figure 1) consists in an illumination source (laser diode), imaging optics (lenses), and a detector (high‐speed camera, CCD, or CMOS). The state‐of‐the‐art is real‐time monitoring with LSP. A thermal or mechanical excitation is essential to activate dynamic processes in the object, though the need for excitations depends on the object's material properties.

**PRACTICAL APPLICATION**

Plant tissue cultures, e.g., hairy roots, produce a spectrum of valuable relevant secondary metabolites. Hairy roots are characterized by a filamentous structure that makes common analytical methods difficult to use [1, 2].

Noncontact measurement with laser speckle photometry (LSP) was used to quantify hairy root cultures. LSP is based on the evaluation of specific interference patterns known as “speckle patterns” [3, 11, 14, 15]. When biological material is illuminated with coherent light, dynamic speckle patterns, known as “biospeckles,” can be observed. Biospeckles contain information about processes modifying an object, and can be used to measure biological and physical phenomena [3, 4, 5, 6]. Time‐resolved biospeckles were used to determine the growth kinetics of the hairy roots. This work proves that LSP is a potential non‐invasive application to determine the growth of *Beta vulgaris* hairy roots and to assess the physical state and growth phase of the cultures in real time.

LSP is based on the detection and analysis of speckle patterns in non‐stationary optical fields. Speckle patterns are random interference patterns formed on a detector and generated by backscattering light when an optically rough object is illuminated with coherent laser light. The speckle patterns are characterized by spatial structures with randomly distributed intensity minima and maxima [9]. The detection of static, dynamic or quasi‐static speckle patterns can be used to characterize material properties, external influences and spatial variation [6, 7, 10, 11]. A static speckle pattern is formed if the laser and the object are stable and the interference pattern does not change over time. The movement of illuminated objects or particles within the object induces an interference pattern that changes over time. These patterns are known as dynamic speckle patterns. As described in Draijer et al., the dynamic behavior of speckle patterns is mainly influenced by Doppler shifts of light interacting with moving particles [12].

If a biological material is illuminated by coherent light, dynamic speckles known as biospeckles can be observed [12, 13, 14]. Biospeckles are interference patterns that change over time and are characterized by a dynamic behavior, induced by physical and biological phenomena related to the object. Biospeckles are formed by backscattering light from the biological material, which is emitted from moving elements of the object, or within the object. The temporal and spatial variation in biospeckle intensity contains information about modification processes affecting the object or occurring within the object and can be exploited to measure biological and physical phenomena [6, 14, 15, 16]. The dynamic behavior of biospeckles is known as biospeckle activity. The dynamic behavior of the illuminated object causes fluctuations, which can be detected in biospeckle images [6, 9, 12].

Elicitors of the dynamic behavior relate to a range of modification processes in cells, e.g., cell division, growth, cytoplasmic streaming, biochemical reactions, or organelle movement. The intensity of biospeckle activity depends on the speed of these processes. The intensity of fluctuation generates blurred areas and reduce the contrast of the speckle images. The contrast of the speckle images can be measured by the parameter of speckle contrast, C [12, 13, 15, 17]. Speckle contrast, C, is defined as the ratio of the SD σ of the intensity I from the mean intensity *I* [9, 12, 18]:

(1) $$C\;=\frac{\sigma }{I}\;=\frac{\sqrt{I^{2}-I^{2}}}{I}\;$$

The speckle contrast can be used to evaluate the biospeckle activity by assessing temporal and spatial speckle fluctuation [12, 19, 20]. The parameter C can have values between 0 and 1. A speckle contrast of 1 indicates an absence of biospeckle activity, or of modification processes. Regions of the highest activity have the lowest speckle contrast [9, 12, 18, 20].

The measurement of the speckle contrast has been used in several applications to quantify characteristics of biological objects and material. The measurement of the perfusion of blood, reported in a variety of studies, was the first achievement using a Laser Speckle‐based technique in medical science [13, 17, 21, 22]. Furthermore, investigations and applications of biospeckle imaging have been carried out to identify biological processes in agriculture [14, 23], to analyze the maturation and bruising of fruits [19, 20, 24, 25, 26], to detect the growth of fungi, parasites, or bacteria, the motility of semen, the viability of seeds [13, 27, 28, 29] or infections on leaf tissues [30] and to quantify plant tissue and root growth [13, 17]. In most studies, the speckle contrast is given as a cumulative value of biospeckle activity and the modification processes are not further differentiated. A few recent reports have described initial approaches to specify biospeckle activity. These studies report on starch and chlorophyll accumulation in apples, thigmostimulation of roots or water transfer in vascular bundles as an origin of biospeckle dynamics [14, 17, 19, 20, 28].

As mentioned before, this work focuses on the evaluation of biospeckles from *B. vulgaris* (hr) measured by a method based on LSP. For the first time, time‐resolved LSP was implemented to assess modification processes occurring in hairy roots to determine the growth kinetics at laboratory scale.

## 2 MATERIALS AND METHODS

### 2.1 Hairy roots of *Beta vulgaris*

Hairy roots of *B. vulgaris* transformed by *Agrobacterium rhizogenis* ATCC 15834 were used as a model organism for all investigations. To induce hairy root cultures, sterile leaves of *B. vulgaris* were infected with the soil bacterium *A. rhizogenes* as described in Pavlov et al. [31]. To infect the leaf, parts of it were wounded so that it exuded phenolic compounds. The bacterium is attracted to the phenolic compounds via chemotaxis and transfers the T‐DNA of a bacterial root‐inducing (Ri)‐plasmid into the chromosomal plant DNA. This infection causes hairy root disease, which induces the cells to proliferate rapidly and form small roots with fine hairs at the infection site [2, 31, 32, 33].

### 2.2 Medium and preculture

To prepare precultures of *B. vulgaris* (hr), Murashige and Skoog solid medium (MS, Duchefa Biochemistry B.V, Haarlem, the Netherlands) supplemented with 30 g/L sucrose (Duchefa Biochemistry B.V) and 5.5 g/L phyto agar (Duchefa Biochemistry B.V) was inoculated with three 15‐mm‐long root tips. The precultures were cultivated for maintenance in standard 92‐mm‐width single‐use Petri dishes (92 × 16 mm, Sarstedt AG & Co. KG, Nümbrecht, Germany) at 26°C in the dark for a period of 21 days. All the samples that were used for the investigations were taken from 3‐week‐old precultures [4, 32, 33, 34].

### 2.3 Preparation of sample cultures

For the experiments, standard 92‐mm‐width single‐use Petri dishes (Sarstedt 821473001) on Murashige and Skoog solid medium (MS, Duchefa Biochemistry B.V, Haarlem, the Netherlands) supplemented with 30 g/L sucrose (Duchefa Biochemistry B.V) and 5.5 g/L phyto agar (Duchefa Biochemistry B.V) were inoculated with four 15‐mm‐long root tips from the preculture. For each experimental running, 40 cultures were prepared, and incubated at 26°C in the dark. Four of the prepared cultures were selected for investigation using Laser Speckle Photometry. The remaining 36 cultures were used to determine the dry weight as a reference value.

### 2.4 Configuration of the laser speckle photometry

The setup for the LSP to capture speckle images from *B. vulgaris* (hr) in solid medium cultures was modified and adapted in preliminary investigations. The configuration of the laser physics (wavelength, power, class), the operating distance, sample position, temperature control, camera settings (exposure time, frame rate, optics (resolution)), and the capturing mode of images (reflection, transmission) were determined experimentally. Disturbances, e.g., scattered light, random reflection, and incident light, were eliminated by covering the setup in a non‐translucent box and fixing the samples to an observation plane. A temperature‐regulating unit was integrated to obtain a constant cultivation temperature of 26°C inside the box and also to prevent the surface of the Petri dishes from fogging up.

The experimental setup was configured as follows. The laser (class 3R, 30 mW, λ = 650 nm, Picotronic GmbH, Koblenz, Germany) was placed over the sample at a distance of 514 mm and irradiated the object at a 5° angle. An optical lens was positioned at a distance of 277 mm in front of the laser to optimize the beam shape and the size of the irradiated area. The speckle images were captured by a camera (UI 336xCP, CMOS Chip 2/3″, 2048 × 1088 pixels, IDS Imaging Development Systems GmbH, Obersulm, Germany) with a variable frame rate in combination with a magnifying lens. The camera was adjusted to a distance of 480 mm and an angle of 23° to the sample.

### 2.5 Detection of biospeckle images

The LSP measurement was started on four 3‐day‐old cultures. Each sample was adjusted in the sample mount at 26°C inside the non‐translucent box and was illuminated constantly by a laser diode. One hundred speckle images of the cultures were captured for each measurement. The exposure time t_ex_ for the image acquisition was configured automatically between 20 and 40 ms. The measurement was repeated every 48 or 36 h for a period of 19 or 31 days, respectively.

### 2.6 Evaluation of biospeckle data

**FIGURE 2:**
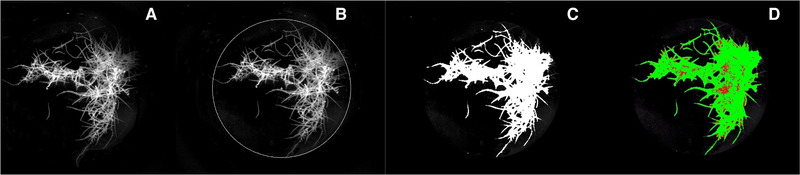
Processing steps with C^#^ software. (A) Biospeckle image, (B) region of Interest (ROI = circle), (C) select the object for assessment, and (D) Result: calculated active areas (green) and non‐active areas (red)

The biospeckle images were evaluated using the image processing C^#^ software with included functions by Halcon® (MVTec Software GmbH, München, Germany) developed by IKTS (Dresden, Germany). The processing steps are illustrated in Figure 2.

Figure 2A shows the captured biospeckle image converted to an 8‐bit grayscale image and Figure 2B presents the region of interest (ROI) selected for evaluation with a circle. The circular ROI including the entire hairy root structure was set manually in the first stage of development then automatically selected using a C^#^ script. In the next step, a threshold was set to label the object for assessment in the ROI (Figure 2C). The time‐resolved biospeckle images were selected, labeled hairy root segments.

### 2.7 Determination of speckle contrast and biospeckle activity

The speckle contrast was computed temporally and spatially according to Equation (1). The calculation was based on laser speckle contrast imaging (LSCI), which is described by Cheng et al. and Draijer [12, 35].

To evaluate the biospeckle activity, thresholds for the speckle contrast were set, which select areas with active and low active behavior. To define the thresholds, the speckle contrast was determined for biospeckle images of single, non‐active hairy root segments (dead) and single, active segments (living). By this means, the biospeckle activity was classified and the selected segments of the biospeckle images were labeled green for active behavior and red for low‐active behavior, as illustrated in Figure 2D. The size of the labeled areas was measured in pixels to assess the distribution of biospeckle activity. The distribution of the biospeckle activity was compared to the reference values that are described below.

### 2.8 Reference methods

At the same time as each LSP measurement, four sample cultures were used to gravimetrically determine the dry weight (m_dw_) as a reference value. In a period of 48 h, the wet biomass of the randomly selected cultures was dried for 48 h at 60°C in balanced Petri dishes ($m_{dw_{\mathrm{plate}}})$. The dry weight was calculated as shown below.

(2) $$\;m_{\mathrm{dw}}=m_{\mathrm{total}}\;-m_{dw_{\mathrm{plate}}}$$

Furthermore, the dry weight was analyzed in order to calculate the specific growth rate (μ [d^−1^]).

(3) $$\mu \left(t\right)=\frac{\ln\left(m_{dw_{2}}\right)-\;\ln\left(m_{dw_{2}}\right)}{t_{2}-t_{1}}\;$$

## 3 RESULTS AND DISCUSSION

**FIGURE 3:**
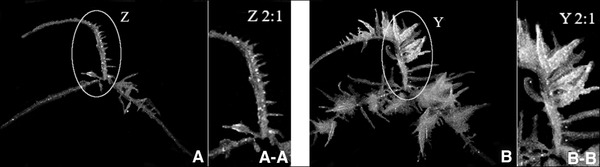
Speckle contrast images of a *B. vulgaris* (hr) culture at 3 days (A) and 5 days (B) old, bright areas in A‐A) illustrate areas with an low speckle contrast and high biospeckle activity, B‐B branches are formed at the bright areas, which are marked in A‐A

The observation of the original 8‐bit biospeckle images of the hairy roots revealed that bright areas have a low speckle contrast. As assumed before, a low speckle contrast indicates high biospeckle activity, and biospeckle activity is induced due to the dynamics of biological processes. Figure 3 shows that bright areas in biospeckle images are characterized by further growth processes [7].

Figure 3B demonstrates that branches are formed at day 5 in segments that are accentuated with bright areas, shown in Figure 3A, 2 days before. The proliferation and cellular differentiation to form branches is caused by a number of intra‐ and extracellular modification processes, e.g., vesicular transport and nutrient uptake [36, 37, 38]. The sum of these biological and physical processes induces high biospeckle activity; therefore, bright areas are specified by a lower *C*.

Consequentially, as a measurement of biospeckle activity, speckle contrast is a parameter to quantify the biomass growth of hairy roots. Subsequently, the biomass growth and the increase in biospeckle activity over the cultivation time is presented.

**FIGURE 4:**
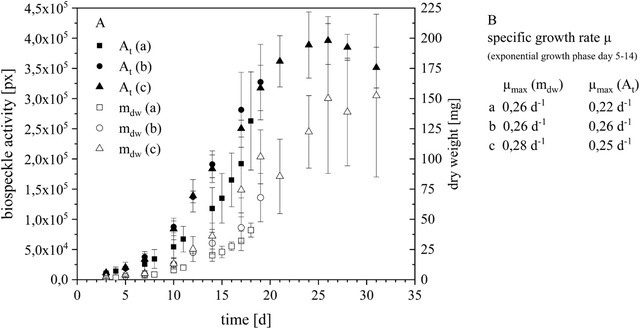
(A) Biomass growth (dry weight m_dw_) of *Beta vulgaris* (hairy roots, 92‐mm‐width Petri dishes, 26°C, Murashige and Skoog medium) and biospeckle activity (*A*_t_) over the cultivation time, *N* = 3 (a,b,c) *n*_a,b,c _= 4, (B): Specific growth rates μ calculated by graphical linear and logarithmic analysis of m_dw_ (t) and A_t_ (t) for exponential growth phase from days 5 to 14

Figure 4A shows the increase in the dry weight *m*_dw_ and the increase in biospeckle activity over the cultivation time *t*. At the beginning of cultivation, the average dry weight *m*_dw_ (*t* = 3) is 1.65 ± 0.83 mg and is detected by an average biospeckle active area *A* (*t* = 3) of 1.02 × 10^4^ *px* ± 2.16 × 10^3^ *px*. After a short lag phase of 2 days, the curves of *A* (*t*) and *m*_dw_ (*t*) rise simultaneously and exhibit exponential behavior. The exponential growth phase is identified between day 5 and day 14 of cultivation. Figure 4B compares the maximum specific growth rates in the exponential phase, which are calculated based on the dry weight or the value for biospeckle activity. The specific growth rates in B vary in a reasonable range between 0.22 per day and 0.28 per day and are comparable to current published specific growth rates for *B. vulgaris* [5].

**FIGURE 5:**
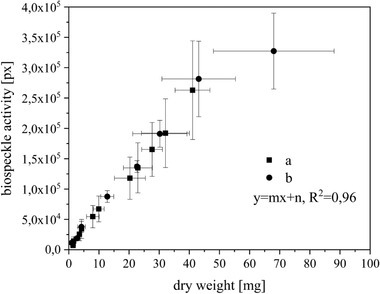
Area of biospeckle activity *A*_t_ measured by speckle contrast as a function of dry weight *m*_dw_, biomass growth of *Beta vulgaris* (Hairy roots, 92‐mm‐width Petri dishes, 26°C, Murashige and Skoog medium)

That means the specific growth rate can also be measured based on the change in biospeckle activity over the cultivation period.

After the exponential growth phase, growth is slow. The dry weight reaches a maximum on day 25 at 150.10 ± 62.04 mg. Concurrent to this, the value of biospeckle activity reaches a maximum of 3.96 × 10^5^ *px* ± 3.96 × 10^4^ *px*. After the maximum level of biomass growth is reached, the stationary phase of biomass growth is achieved and the value of dry weight stays constant. However, at this point the biospeckle activity curve provides more information about the biomass characteristics. The distribution of biospeckle activity decreases after reaching the maximum. As mentioned before, the biospeckle activity corresponds to modification processes occurring in the cells. Accordingly, if the biospeckle activity decreases, then the dynamic in the cells also declines. On this assumption, the necrosis of the cells is also monitored by the biospeckle activity.

The biospeckle activity can be used to quantify the biomass growth of *B. vulgaris* (hr). The biospeckle activity changes according to the cell dynamics and correlates to the dry weight increase. The correlation of *A*_t_ (*m*_dw_) is illustrated below.

The correlation between *A*_t_ and *m*_dw_ is identified as a curve with substantially differentiated parts. From the beginning of cultivation to a dry weight of approximately 40 mg, a linear increase is detected with a coefficient of determination *R* ^2 ^= 0.996. This linear behavior accompanies the lag and exponential growth phases. After the exponential growth phase (40 mg), the graph increases more slowly due to the lower change in biospeckle activity, which corresponds to the reduced growth of biomass. Because of the heterogeneous structure of hairy roots, the deviation in the values increases during the development stage. The maximum is reached at 150 mg (dw), as shown above in Figure 3, and the biomass growth reaches the stationary phase at a certain point. In the stationary phase (Figure 4), the value for *A*_t_ (*t*) decreases asymptotically at the constant maximum of *m*_dw_.

In summary, the biomass growth of *B. vulgaris* (hr) can be quantified by the biospeckle activity. The change in biospeckle activity correlates to the increase in the dry weight. The substantially differentiated parts of the correlation *A*_t_ (*m*_dw_) can be summarized as linear behavior until the end of exponential growth, a slow increase until the maximum of m_dw_ is reached and an asymptotic decrease at the constant maximum m_dw_.

Furthermore, the values for A_t_, the total area of biospeckle activity, can be more differentiated. *A*_t_ was divided into active *A*_a_ [px] and low‐active areas *A*_l_ [*px*]. The spatial distribution of biospeckle activity was labeled as active parts (green) and low‐active parts (red). The distribution of biospeckle activity for one representative culture is shown below.

**FIGURE 6:**
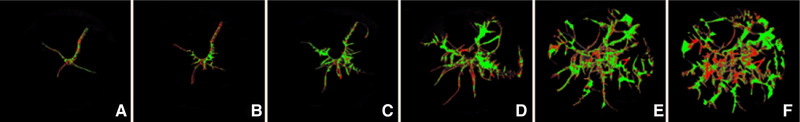
Spatial distribution of biospeckle activity measured by speckle contrast classified as active areas (green) and low active areas (red) from *Beta vulgaris*, hairy roots in 92‐mm‐width Petri dishes, 26°C, Murashige and Skoog medium, after a cultivation time of (A) 3 days, (B) 5 days, (C) 7 days, (D) 10 days, (E) 14 days, (F) 17 days

Figure 6 represents the distribution of biospeckle activity in a heterogeneous hairy root culture during cultivation. The spatial distribution of the biospeckle activity is seen to shift. At the beginning of cultivation, active and low‐active areas are spread randomly and are equal in dimension. Furthermore, active behavior dominates, synchronized to the exponential growth phase (5–14 days). At the end of the cultivation assay from day 17, significantly less active behavior is detected in the center of the hairy root culture and active areas are located in the outer regions.

**FIGURE 7:**
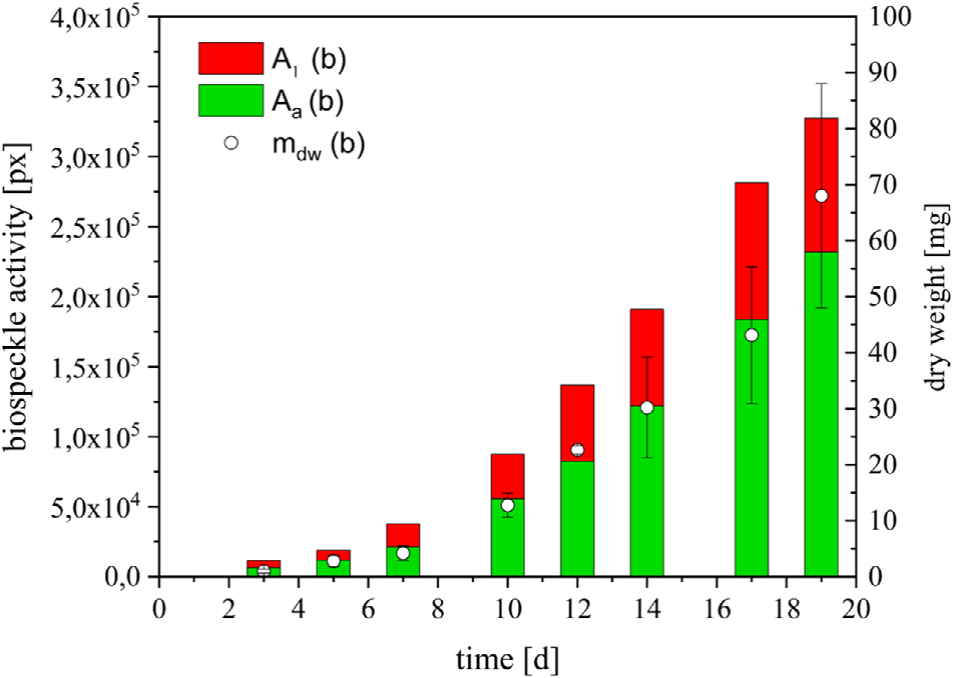
Distribution of biospeckle activity measured by speckle contrast and classified as active areas (green) and low active areas (red) from *Beta vulgaris*, hairy roots in 92‐mm‐width Petri dishes, 26°C, Murashige and Skoog medium, and the dry weight (m_dw_) over the cultivation time

As described above, the biospeckle activity is related to the biological and the physical dynamics of the object. The distribution of the biospeckle activity reflects the observation of biomass growth in Petri dishes. Branches in the center of the hairy root culture are older and their nutrient supply is limited due to confinement. Besides that, the growth of hairy roots is characterized by elongation of the tips. The dynamic behavior in root tips and in younger segments is indicated by the green areas. This visualization of the distribution of biospeckle activity in hairy root cultures is innovative and a progressive method to describe the state of the culture's development. The following Figure 7 presents an alternative illustration of the distribution of biospeckle activity compared to the biomass growth from the same culture as shown in Figure 6.

Figure 7 presents the distribution of biospeckle activity of one representative sample culture compared to the biomass growth. The columns illustrate the increase in biospeckle activity A_t_, which is divided into a green part for active A_a_ and a red part for less active A_l_. As described above (Figure 3), the increase in biospeckle activity and the increase in dry weight took place simultaneously. Figure 7 illustrates the lag and the exponential growth phases. The focus in this illustration is on the distribution of biospeckle activity. As shown and described in Figure 6 above, at the beginning, in the lag phase, the red and green parts are almost equal. During the exponential growth, the active part increases more than the low‐active part. At the end of the exponential growth, significantly more active areas are detected. Conversely, this means that at this point of cultivation, the cultures are at a highly dynamic level and a large number of modifications are in process.

In conclusion, the evaluation of the biospeckle activity provides a variety of information about the object of interest. The biomass growth of hairy roots can be determined. The state of development of the biomass growth is characterized by the biospeckle activity. Furthermore, in the stationary phase, the biospeckle activity still gives indications about the dynamic of biological and physical phenomena of the object or in the cells. Besides this, the biospeckle activity of the culture is further divided into more differentiated in low active and active areas. The distribution of the biospeckle activity is visualized as a spatial and time‐resolving parameter. The biospeckle activity calculated by means of the speckle contrast is a useful parameter for quantifying hairy roots.

The speckle contrast reflects the sum of modification processes, but the difference between the biospeckle activities is currently not further differentiated into active and low‐active behavior. However, the dynamic behavior of biospeckle images from hairy roots of *B. vulgaris* can be affected by a variety of physical and biochemical processes, e.g., by branching effects, elongation, secondary metabolite accumulation, cytoplasmic streaming, cell organelle movement, nutrient uptake, diffusion, Brownian motion, and transfer processes [5, 32, 34, 39, 40]. The interpretation and further classification of biospeckle activity is therefore difficult, but purposeful and useful for further applications. Advanced investigations on the micro‐ and nanoscale are a practical means of identifying biospeckle activity induced by processes in hairy roots.

The further development of application‐oriented LSP focuses on liquid cultures, especially for cultivation in shake flasks and bioreactors. In other studies by the authors, LSP has also been successfully applied to liquid cultures. Due to some optical disturbances, further optimizations are required.

Nevertheless, this work demonstrates the successful implementation of LSP to assess hairy root cultures.

The growth kinetics, growth phase and physiological behavior of *B. vulgaris* (hr) can be detected and evaluated using LSP. LSP measurement takes only a few milliseconds, which means a saving of 48 hrs compared to the reference method. LSP is an simple optical, automated and rapid method with an affordable setup.

The evaluation of biospeckle images is a means of gaining comprehensive temporal and spatial information about the objects of interest. Biospeckles provide an overall assessment of the sample and physiological states [14].

## 4 CONCLUDING REMARKS

LSP is a potential tool for monitoring biotechnological processes, which is indispensable for understanding, optimizing and simulating production processes. Plant cells and tissue cultures, e.g. hairy roots, produce a wide range of nutritionally, physiologically and pharmaceutically relevant secondary metabolites. Hairy roots are characterized by a filamentous structure, which makes common analytical methods difficult to apply [2, 3]. Due to this fact, LSP was implemented to characterize the biomass growth of hairy roots cultures from *B. vulgaris*. Biospeckle images were evaluated using speckle contrast analysis to measure the biospeckle activity. The biospeckle activity corresponds to processes modifying the object and correlates with the biomass growth. The increase in biospeckle activity occurs at the same time as the increase in biomass. The different growth phases (lag, exponential, and stationary) are monitored by observing the biospeckle activity during the cultivation. The maximum specific growth rates of all experiments calculated based on the change in biospeckle over the cultivation time are equivalent. The curve of the biospeckle activity as a function of the dry weight is linear until the end of exponential growth, then increases more slowly and ends with asymptotic behavior at the maximum dry weight. Besides this, after the biomass growth enters the stationary growth phase, the biospeckle activity decreases, which reflects the aging process of the cells. Furthermore, based on the distribution of the biospeckle activity, segments of the culture are divided into parts with active and low active behavior. The distribution of the biospeckle activity enables the cultures’ physical state to be evaluated, which is a completely new approach.

As an innovative, non‐invasive, non‐destructive, rapid method that can be used online, LSP is a potential application to determine the growth of hairy root cultures of *B. vulgaris* and to assess the cultures’ physical state or growth phase.

The speckle contrast as an index for biospeckle activity is currently classified for *B. vulgaris* (hr) in active and low‐active spatial distribution. For commercial use, the LSP technique and biospeckle method require an accurate interpretation of biospeckle phenomena [14]. The modification processes that are responsible for biospeckle activity must be identified.

Further investigations on the micro‐ and nanoscale are required to achieve this.

Nevertheless, the LSP technique is a suitable and sensitive analytical method to determine the biomass growth of hairy roots in solid agar cultures. The time required to determine the biomass growth is significantly reduced and the optical setup is very simple and portable compared to existing methods. LSP is an approach that provides an overall assessment of the sample and physiological states.

*The authors are grateful for the financial support of this work by the European Regional Development Fund (ERDF) and the Free State of Saxony (project number: 100187245)*.

*The authors have declared no conflict of interest*.

## NOMENCLATURE

**Table:** 

A	[px]	area
C	[‐]	speckle contrast
I	[mW]	intensity
m	[mg]	mass
t	[ms, min, h, d]	time
*Greek symbols*		
σ	[‐]	standard deviation
μ	[d^−1^]	specific growth rate
*Indices*		
a		active
dw		dry weight
l		low active
max		maximum
plate		Petri dish
total		total
exp		exponential
ex		exposure
